# PedHunter 2.0 and its usage to characterize the founder structure of the Old Order Amish of Lancaster County

**DOI:** 10.1186/1471-2350-11-68

**Published:** 2010-05-02

**Authors:** Woei-Jyh Lee, Toni I Pollin, Jeffrey R O'Connell, Richa Agarwala, Alejandro A Schäffer

**Affiliations:** 1 National Center for Biotechnology Information, National Library of Medicine, National Institutes of Health, 8600 Rockville Pike, Bethesda, Maryland 20894, USA; 2 Department of Medicine, Division of Endocrinology, Diabetes and Nutrition, University of Maryland School of Medicine, 660 W. Redwood Street, Baltimore, Maryland 21201, USA; 3 Animal Improvement Programs Laboratory, Agricultural Research Service, United States Department of Agriculture, 10300 Baltimore Avenue, Beltsville, Maryland 20705, USA

## Abstract

**Background:**

Because they are a closed founder population, the Old Order Amish (OOA) of Lancaster County have been the subject of many medical genetics studies. We constructed four versions of Anabaptist Genealogy Database (AGDB) using three sources of genealogies and multiple updates. In addition, we developed PedHunter, a suite of query software that can solve pedigree-related problems automatically and systematically.

**Methods:**

We report on how we have used new features in PedHunter to quantify the number and expected genetic contribution of founders to the OOA. The queries and utility of PedHunter programs are illustrated by examples using AGDB in this paper. For example, we calculated the number of founders expected to be contributing genetic material to the present-day living OOA and estimated the mean relative founder representation for each founder. New features in PedHunter also include pedigree trimming and pedigree renumbering, which should prove useful for studying large pedigrees.

**Results:**

With PedHunter version 2.0 querying AGDB version 4.0, we identified 34,160 presumed living OOA individuals and connected them into a 14-generation pedigree descending from 554 founders (332 females and 222 males) after trimming. From the analysis of cumulative mean relative founder representation, 128 founders (78 females and 50 males) accounted for over 95% of the mean relative founder contribution among living OOA descendants.

**Discussion/Conclusions:**

The OOA are a closed founder population in which a modest number of founders account for the genetic variation present in the current OOA population. Improvements to the PedHunter software will be useful in future studies of both the OOA and other populations with large and computerized genealogies.

## Background

The *Old Order Amish (OOA) *of Lancaster County in Pennsylvania are a closed founder population, with approximately 40,000-50,000 living individuals that can be connected into a single 14-generation pedigree. Other large OOA populations live in Ohio and Indiana. There have been numerous medical genetics studies on the OOA. For several decades, the medical genetics studies focused on monogenic diseases such as brittle hair disease [1,2].

More recently, research interests have broadened to include complex traits, such as Parkinson disease [3,4], dementia [5], diabetes [6], blood pressure [7,8], hip fractures/osteoporosis [9], and vision phenotypes [10]. Investigators at the University of Maryland School of Medicine (UM-SOM) [11] have recruited over 4,800 OOA from Lancaster County for several studies of complex adult-onset diseases beginning in 1993 with the initiative of Dr. Alan R. Shuldiner [6,12]. These OOA studies have used genome-wide linkage analysis [13-15], candidate gene association analysis [16], and most recently genome-wide association studies (GWAS) to discover variants influencing traits such as cardiac repolarization [17], type 2 diabetes [18], hypertension [19], and fasting and post-prandial triglyceride levels [20] or to validate locus associations first discovered in other populations, e.g., diabetes [21], human height [22], fasting glucose levels [23], waist circumference [24], bilirubin levels [25] and response to the anti-clotting agent clopidogrel [26].

The OOA comprise one of several groups in the more general category of Anabaptists; other Anabaptist groups living in North America include other Amish, Mennonites, and Hutterites. Medical geneticists have been interested in Anabaptist populations because they are closed populations and have written genealogies and other features such as a homogeneous, rural lifestyle and high standard of living [27-29]. Their genealogies were mostly published in paper books, which make integration and search challenging and time-consuming tasks.

In 1996 we set out to construct a computer-searchable genealogy database of the OOA of Lancaster County in Pennsylvania for use by geneticists [30]. To support the database, we developed a suite of query software that would solve pedigree-related problems automatically and systematically. The digital genealogy database expanded to include other Anabaptist populations and was renamed *Anabaptist Genealogy Database (AGDB) *[31-33], and the query software package was named *PedHunter *[30,33]. The initial source to construct AGDB was a 1996 computer file updating the *Fisher Family History (FFH) *book [34] edited by Ms. Katie Beiler. Later versions integrated the *Amish and Amish Mennonite Genealogies (AAMG) *book [35], a large computerized genealogy file from Mr. James Hostetler and two smaller updates from Ms. Beiler of recent births, deaths, and marriages in the Lancaster area. The queries and utility programs of PedHunter are illustrated by examples using AGDB in this paper.

Several groups have used AGDB in their research. Some worked on rare diseases, such as nemaline myopathy [36], congenital microcephaly [37], and dystonia [38,39]. Although AGDB contains no explicit phenotype data, lifespan can be inferred when both birth and death dates are available. Several studies on lifespan using AGDB have established that lifespan is heritable [40], that lifespan may be associated with other implicit traits [41,42], and that lifespan can be associated with experimentally measured traits [43].

A frequent problem in medical genetics is to reconstruct the pedigree relationships among distant relatives. PedHunter was first designed to solve optimal pedigree connection problems and other problems related to pedigree construction, verification, and analysis. PedHunter is not formally tied to AGDB; in particular, access to AGDB requires ethics approval from an Institutional Review Board (IRB), while PedHunter is freely available. PedHunter has been used to construct genealogies for linkage and haplotype analysis on Hutterite families [44-47] and analyses of an Icelandic population [48], a Southern Italy population [49], and a Northern Italy population [50].

In this paper, we report on new features in PedHunter and on how those features can be used to quantify the impact of the pedigree structure of a founder population on the amount of genetic variation present in the current population. Under a model of genetic drift, the number of founder alleles present in the current population will depend on factors such as sibship size and number of new genomes that enter each generation. From AGDB we can answer this question: How many OOA founders contributed what expected percent of the genetic material to the present-day living OOA? The answer impacts the genetic architecture, and hence phenotypic distribution of important traits such as low density lipoprotein (LDL) and triglyceride (TG) levels. A recent study characterized the patterns of linkage disequilibrium in the OOA [51]. In this paper, we quantify the representation of founder genes in the current population that contribute to those patterns of linkage disequilibrium.

## Methods

### PedHunter Versions

PedHunter version 1.0 [30] that provided 23 queries was first released in 1998. In versions 1.1, 1.2, 1.3, and the current 2.0 we increased the number of queries to 50. The full list of 50 query programs and seven utility programs is in the Additional file 1. PedHunter 2.0 has been tested on platforms running the Linux, SunOS, Windows, and Mac OS X operating systems. The queries are implemented using Transact-SQL and C version of Open Client DB-Library. All PedHunter queries and utility programs are available as executable files that can be used from the command line prompt and in UNIX shell scripts. The current version of PedHunter is freely available and can be downloaded from http://www.ncbi.nlm.nih.gov/CBBresearch/Schaffer/pedhunter.html.

PedHunter queries a genealogy database stored either as a SYBASE relational database or in structured ASCII plaintext files. Before version 2.0, the source codes for these two variants were separate with much duplication. The single set of code files in version 2.0 can be adapted to either type of database representation with small changes to one code header file.

### Queries in PedHunter 2.0

PedHunter 2.0 supports four categories of queries as basic operations: 1) testing a relationship; 2) finding all individuals satisfying a certain relationship; 3) printing information; and 4) complex queries. We use italics to indicate the names of specific queries. Queries that find pedigrees print the pedigrees in LINKAGE format [52].

In the second category, PedHunter 2.0 adds two queries pertinent to our study of OOA founder structure: *founder_descendant *to find founders of a given individual, and a new query *count_descendant *to count number of descendants per ancestor from an input file in the format of ancestor-descendant pairs. New complex queries pertinent to our study include: *calculate_r *to calculate relative founder representation (RFR) for a founder-descendant pair, and *average_r *to calculate mean RFR per founder in AGDB, as explained below.

### Examples of Queries Useful for Analysis of the Anabaptist Genealogy Database

To find possible participants in AGDB to be recruited into a study, the *living *query is useful to find living individuals. The *founder_descendant *query can be followed to find the founders of each living individual. For each founder-descendant pair, the *calculate_r *tool computes the RFR, defined below. The *average_r *tool then computes the mean RFR for each founder. The person information for founders can be obtained using the *person_info *query. Prior to running the *asp *query to find "all shortest paths" pedigree(s) connecting a set of sampled individuals, the *subset *query is required to first find a maximal subset of individuals that shares a common ancestor.

### Pedigree Renumbering

PedHunter is also useful for genetic evaluations on large pedigrees. For example, at the United States Department of Agriculture (USDA), the number of animals in the Holstein dairy pedigree used for genomic evaluation of important economic traits has grown from 41,000 to 125,000 in less than 18 months as new animals are added. The ability of PedHunter to scale to millions of subjects is a feature. Other pedigree storage/query packages, such as PEDSYS [53], are not able to process such a large pedigree (though PEDSYS adds the capability to connect large amounts of phenotype data with individuals in smaller pedigrees, such as the subset of more than 4,800 OOA individuals that have been studied at UM-SOM). To facilitate handling large pedigrees, we added the *renumber_pedigree *utility to PedHunter to number and order subject identifiers, and add missing spouses/mates if necessary into the pedigree. The renumbering process ensures the property that identifiers of parents are smaller than the identifiers of their children, which enables more efficient calculation of kinship coefficients [[54], Ch. 5]. Adding missing parents into the pedigree is necessary for some software packages, such as LINKAGE, which assume that each person has either zero or two parents in the pedigree. The original identifiers are included in the output, so that information can still be connected to these identifiers.

### Anabaptist Genealogy Database Version 4.0

AGDB was created in SYBASE SQL Server release 11.0.x of the SYBASE relational database management software. Each individual in AGDB is assigned a unique integer, called the *program id*. Two main tables in the PedHunter pedigree storage system are the *person *table and the parent-child *relationship *table.

There have been several versions of AGDB based on the sources mentioned in Background and with sizes summarized at the beginning of Results. The current AGDB version 4.0 (AGDB4) was created in 2004, after the most recent published description [33], to combine all the sources and updates. Some small batches of corrections and updates have been incorporated based on feedback from users.

As indicated by the change in the first word of AGDB from the original "Amish" to "Anabaptist", many of the individuals in AGDB are not OOA. As explained in Results, we extracted a subpedigree from AGDB4 that should include most living OOA individuals in the Lancaster area and their ancestors. We used the method described in the next subsection to predict which individuals are OOA.

### Prediction of Old Order Amish Status

An adult or near-adult (older adolescent) is of OOA status if the individual belongs to the OOA church, which means that the individual has chosen to be baptized. For the purpose of our query, we included children and young adults who were not yet baptized but lived with their OOA parents. For individuals in the printed FFH book, determining the OOA status by eye is usually easy, because for most family records, the religious affiliation is included. However, there are some omissions and some cases that are unclear. Due to the syntax in FFH, the word "OOA" appeared only once per family record. When AGDB was generated, the OOA status was captured only into the record of the *person *whose name preceded the word "OOA". If the family head was married, the OOA status was usually assigned to the spouse of family head in AGDB. For example, the FFH book reads "1. Christian Fisher b April 26, 1757, d Nov. 19, 1838, farmer, m Barbara Yoder (Yost Yoder) OOA, ...". Christian Fisher's *person *record in AGDB does not include any OOA status, but Barbara Yoder's record does. The spouse and children of the person assigned OOA status should also have OOA status in most cases. Therefore, to predict if an individual in the genealogy requires some inferences across *relationship *links. For individuals added into AGDB by Ms. Beiler after the FFH book was printed, the amount of information about religious affiliation rapidly declined to zero. We believe that virtually all individuals added in updates by Ms. Beiler in 1999 and 2003 are OOA, due to biased ascertainment.

Prediction of OOA status is done in three different ways: 1) for an individual born in or before 1971, we consider *person *records of self, parents and spouses; 2) for an individual born in 1972-1986, we check *person *records of self, parents, spouses and spouses' parents; 3) for an individual born in 1987 or later, we examine *person *records of self, parents, grandparents and spouses. For an individual *I*, if any of the relatives of *I *considered has OOA status, then we predict that *I *has OOA status.

### Pedigree Trimming on Founders

**Figure 1:**
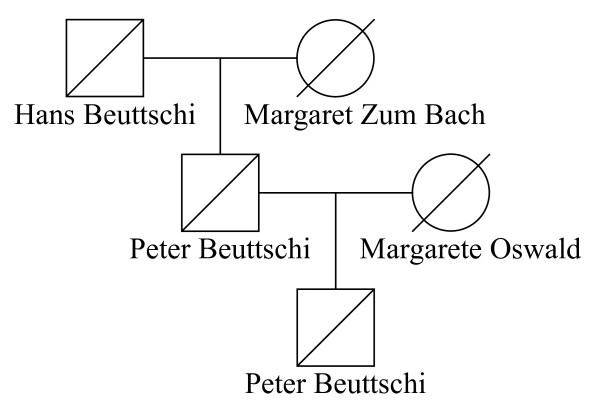
**Sample pedigree trimming in AGDB4**. Two recursive rounds of pedigree trimming on three founders down to one *trimmed *founder in AGDB4.

Working with large pedigrees, trimming redundant or irrelevant individuals (as defined below) from a pedigree will reduce the computational time of some pedigree analysis methods [55]. In a nuclear family that is at the top of the pedigree and has only one child, we can replace both parent founders with their only child in the analysis because such a child's genomic data represents all genomic data derived from those parents. Nuclear families at the top of pedigree are recursively trimmed, until no more trimming is possible. Figure 1 illustrates a married founder couple Hans Beuttschi and Margaret Zum Bach with their only son Peter Beuttschi. After one round of trimming, Peter Beuttschi replaces his parents as a *trimmed *founder. Peter Beuttschi was married to Margarete Oswald, who is a female founder, and gave birth to their only son also named Peter Beuttschi. After a second round of trimming, Peter Beuttschi replaces his parents as a new *trimmed *founder.

### Estimation of Birth Years for Founders

Many of the older *person *records in AGDB did not have birth dates in the data sources. To report our analysis by birth cohorts, it is necessary to estimate birth years for individuals; the estimated birth years are used for the analysis in this project, but are not recorded within AGDB. For simplicity and to allow inclusion of records with known dates missing the month or day, we used only birth *years *but not actual birth *dates*. To systematically estimate missing birth years, we performed a frequency test on known *parent-child *pairs with known birth years for both individuals in AGDB4. We extracted birth year of each founder record with known birth year, and subtracted from birth year of the oldest child with known birth year. There are 7,373 known founder-and-oldest-child pairs. The mean birth year difference is "25.7 years", and the median birth year difference is "25 years". Therefore, we used 25 years per parent-child generation in the estimation of birth years for founders in AGDB4.

If the birth year of a founder was unavailable, we subtracted 25 years from the birth year of the (known or estimated) oldest child. If birth year of a child is unavailable, we recursively estimated birth years among the children of the child. The following example estimates a founder (unknown name in AGDB4)'s birth year to be "1695" by subtracting 75 years (three generations) from the estimated oldest child (Henry Stehly)'s estimated oldest child (Magdalena Stehly)'s oldest child (Catherine Sieber) who was born in 1770.

### Mean Relative Founder Representation

We created and used the *calculate_r *tool to calculate the relative representation of each founder in each study participant. We define the RFR of a given founder in a given descendant as the expected proportion of alleles in the descendant that were inherited identical-by-descent (IBD) from the founder. For example, an offspring inherits half its genome from each parent, thus the founder representation for each parent is one-half. The expected proportion can be computed as twice the kinship coefficient between the parent and offspring. The kinship coefficient is defined as the probability at a given locus that the allele selected from one individual will be identical by descent to a randomly selected allele of a second individual. For example, twice the kinship coefficient between a parent and an offspring, and the RFR of that parent in that offspring, is 0.5. In a three-generation pedigree, the grandparent-grandchild kinship coefficient is 0.125; thus the relative representation of each grandparent in a grandchild is 0.25. It should be noted that founder representation represents the average across the genome, whereas at any autosomal locus the two alleles an individual inherits come from at most two founders. From these definitions and the pedigree structure we use *average_r *to calculate the mean RFR for each founder over all study descendants. By definition the RFRs in a given individual will sum up to 1.

A subtle point is that the founder representation (expected IBD) as described above is calculated assuming no inbreeding in the founders because by definition, their ancestors are not in the genealogy. Conceptually, the probability of inheriting a founder allele is a property of Mendelian sampling in the pedigree, independent of the probability that the two alleles of a founder are inherited IBD from unknown ancestors. Thus, the RFR of a parent in an offspring is 0.5 only under the assumption of no inbreeding. If the parent were completely inbred (say a mouse strain) then twice the kinship coefficient would be 1.0. The former agrees with what we expect, while the latter would imbalance the founder representation between an inbred father and non-inbred mother.

## Results

The size of AGDB has grown from AGDB1, completed in 1998 containing 55,636 individuals and 12,896 marriages, to the current AGDB4, containing 417,789 individuals and 102,341 marriages [30-32]. In 2003, we built a special version called FISHER that includes 66,131 individuals and 15,057 marriages to accommodate three updates since FFH was published in 1988. The results here are based on both FISHER and AGDB4. The subjects of interest in the ongoing genetic studies at UM-SOM are in FISHER, but some of their ancestors who are not in FISHER can be found in AGDB4.

### Distribution of Old Order Amish

**Figure 2:**
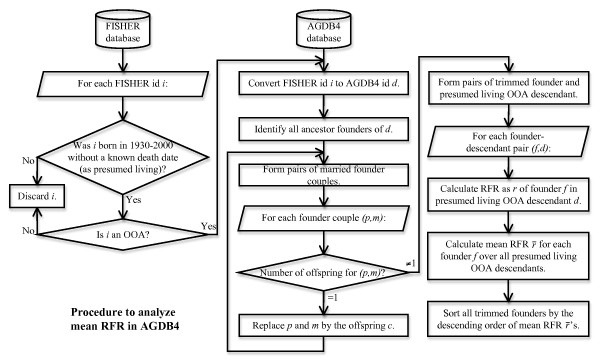
**Procedure to analyze mean RFR in AGDB4**. Flowchart to analyze mean RFR of founders in presumed living OOA descendants.

The procedure to analyze mean RFR of founders in presumed living OOA individuals is illustrated in Figure 2. By executing the *age *tool in PedHunter 2.0 on the 66,131 individuals in FISHER, we collected 54,411 individuals born in 1930-2000. Using the *living *tool in PedHunter 2.0, we found 51,905 presumed living individuals, with no death date recorded in FISHER. By predicting the OOA status among 54,411 individuals born in 1930-2000, we find 36,057 to be OOA. Among 51,905 presumed living individuals, the results showed 34,160 presumed living OOA individuals born in 1930-2000 and in FISHER. The difference between the 34,160 individuals used here and the 40,000-50,000 estimate in the Background is due to children born in 2001-2009 and individuals born before 1930 who are still alive. We focused attention on the individuals born in 1930-2000 because death records for those born before 1930 are rather incomplete, and birth records after 2000 are currently incomplete, although we plan to address these deficiencies in AGDB.

Table 1 reports the birth year and gender distribution among above 34,160 presumed living OOA individuals in FISHER. The results show increasing numbers of births over time. The year with the most newborns is 1995, in which there were 1,073 birth records (488 females, 572 males, and 2 of unknown gender). The data consistently show more males than females born.

**Table 1:** Distribution of birth years and genders for presumed living OOA in FISHER.

Birth year	Female	Male	Unknown gender	Sum
**1930-1940**	651	650	0	1,301
**1941-1950**	1,014	1,128	0	2,142
**1951-1960**	1,360	1,452	0	2,812
**1961-1970**	1,894	2,042	0	3,936
**1971-1980**	3,131	3,320	0	6,451
**1981-1990**	4,031	4,347	4	8,382
**1991-2000**	4,414	4,698	24	9,136

**Total**	16,495	17,637	28	34,160

Birth year cohorts of ten years are reported in rows, and genders in three categories are reported in columns. The total number of presumed living OOA individuals in FISHER is 34,160.

We then located these 34,160 individuals in AGDB4 by mapping their *program ids *from FISHER to AGDB4. Using the *ancestors *tool in PedHunter, we constructed a pedigree containing a total of 43,162 individuals, including the above 34,160 presumed living individuals, in AGDB4. The 43,162 individuals form a 14-generation pedigree, in which there are 606 founders. There are 22 living individuals recorded as founders themselves. The OOA study at UM-SOM confirms that many of these are spouses of individuals who left the Amish church, although they may have been OOA at some time and may have OOA children.

Among the 606 founders, 355 are female and 251 are male. There are 175 marriages between 175 female founders and 170 male founders; five male founders had two marriages to female founders, with the second marriage following the death of the first spouse in all cases. Among 175 marriages, 102 founder couples have exactly one child. By applying the trimming technique described in Methods, 606 founders are reduced down to 555 *trimmed *founders after one round and 554 *trimmed *founders after two rounds. All subsequent analysis of founders uses this trimmed set of 554 founders. In the trimmed set, there are 332 female founders and 222 male founders, and 136 married founder couples including 136 females and 131 males.

Table 2 reports the birth years for the 554 founders. The two most ancient founders are a married couple, estimated to have been born in 1670. Prior to 1776, birth years of more than 50% of founders are not recorded in AGDB4. All but one founder born after 1866 have known birth years.

**Table 2:** Distribution of known and estimated birth years of 554 founders in AGDB4.

Birth year	Female	Male	Sum
**1666-1675**	1 (1)	1 (1)	2 (2)
**1676-1685**	8 (7)	8 (7)	16 (14)
**1686-1695**	5 (5)	4 (4)	9 (9)
**1696-1705**	13 (12)	14 (8)	27 (20)
**1706-1715**	19 (18)	10 (8)	29 (26)
**1716-1725**	15 (11)	11 (6)	26 (17)
**1726-1735**	21 (17)	12 (7)	33 (24)
**1736-1745**	23 (18)	15 (8)	38 (26)
**1746-1755**	27 (22)	14 (8)	41 (30)
**1756-1765**	17 (14)	14 (6)	31 (20)
**1766-1775**	18 (11)	9 (4)	27 (15)
**1776-1785**	22 (10)	13 (5)	35 (15)
**1786-1795**	19 (6)	15 (5)	34 (11)
**1796-1805**	19 (7)	15 (2)	34 (9)
**1806-1815**	14 (2)	11 (5)	25 (7)
**1816-1825**	10 (2)	8 (4)	18 (6)
**1826-1835**	13 (3)	13 (3)	26 (6)
**1836-1845**	8 (4)	4 (2)	12 (6)
**1846-1855**	6 (1)	4	10 (1)
**1856-1865**	4 (2)	7 (3)	11 (5)
**1866-1875**	4	3 (1)	7 (1)
**1876-1885**	4	1	5
**1886-1895**	1	1	2
**1896-1905**	6	0	6
**1906-1915**	5	1	6
**1916-1925**	2	1	3
**1926-1935**	2	2	4
**1936-1945**	1	0	1
**1946-1955**	6	1	7
**1956-1965**	9	2	11
**1966-1975**	5	8 (1)	13 (1)
**1976-1985**	5	0	5

**Total**	332 (173)	222 (98)	554 (271)

Birth year cohorts of ten years are reported in rows, and genders are reported in columns. The numbers represent both known and estimated birth years in the age group; the numbers in parentheses are the number of founders among that cohort whose birth years were estimated, as described in Methods.

### Old Order Amish Founder Structure

Using the pedigree structure from AGDB4, one can estimate the contribution of the founders to the current OOA gene pool. This is an estimate because, as described in Methods, the calculations are based on probabilistic analysis of the pedigree structures rather than observed DNA segregation. Moreover, there may be additional relationships (not in the genealogy) among the designated founders and some relationships in the genealogy may be inaccurate. Table 3 reports on the birth years of 554 founders and numbers of their living OOA descendants. The largest coverage number is 34,100 living descendants, who are descended from the oldest founder couple. Of 554 founders, 361 were born before 1800. However, many of these founders had few descendants. We were interested in calculating the number of founders contributing to the majority of the extant population to evaluate qualitatively and quantitatively our assumptions about the utility of the OOA for gene mapping.

**Table 3:** Distribution of birth years and numbers of descendants among 554 founders in AGDB4.

Birth year	1≤#(D)<10	10≤#(D)<100	100≤#(D)<1,000	1,000≤#(D)<10,000	10,000≤#(D)	Sum
**1666-1715**	0	0	3	30	50	83
**1716-1765**	8	23	58	54	26	169
**1766-1815**	17	51	66	19	2	155
**1816-1865**	24	29	19	4	1	77
**1866-1915**	9	10	7	0	0	26
**1916-1965**	25	1	0	0	0	26
**1966-1985**	18	0	0	0	0	18

**Total**	101	114	153	107	79	554

Birth year cohorts of fifty years are reported in rows, and numbers of descendants #(D) are reported in columns. Founders born earlier have larger numbers of presumed living OOA descendants.

We used the *calculate_r *and *average_r *tools in PedHunter 2.0 to calculate the relative representation of each founder in an average living OOA individual. By ranking founders in descending order of their founder representation, we were able to count the number of founders represented in the majority of expected alleles in an average individual among living OOA individuals.

**Figure 3:**
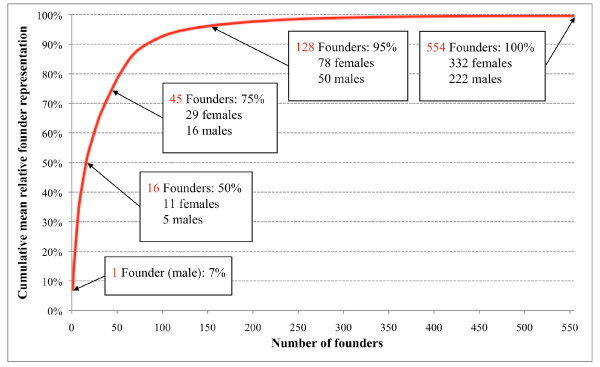
**Cumulative mean RFR in AGDB4**. Cumulative mean RFR of 554 founders in 34,160 presumed living OOA descendants.

We computed the average expected genetic contribution of each of the 554 founders (332 females and 222 males) to each of the 34,160 presumed living OOA descendants as summarized in Figure 3. We found that 128 founders (78 females and 50 males) accounted for over 95% of the average founder contribution, with a single founder accounting for nearly 7% of the average contribution. Half of the total founder representation came from only 16 founders (11 females and 5 males). These data quantify the extent to which the OOA are a closed founder population ideal for elucidating the role of genetic variation in complex diseases.

To put the coverage analysis in some chronological perspective, it is useful to consider Isaac Huyard who was born on February 7, 1865. He married into the OOA on December 8, 1891 after working as a servant for an Amish family, and is anecdotally the last individual who was born and reached adulthood non-OOA, and then became OOA [56]. 125 of the highest contributing 128 founders (accounting for 95% of gene pool) were born in or before 1865. The other three high-contributing founders were born in 1867, 1901 and 1904. On the other hand, 70 founders born after 1865 account for only 0.8% of the founder representation. Thus, a detailed study of the pedigree structure of the OOA as catalogued in AGDB supports the notion that there has been very little influx of genetic material into the OOA population since the mid-19th century.

Among 70 founders born after Isaac Huyard, we found that 12 founders are adopted, and another two founders are likely adopted. One of the improvements in going from AGDB3 to AGDB4 was the identification of adopted individuals, so that those "non-biological parent to child" links are not in the pedigrees that we construct, if the adoptive relationships are known.

### Relevance of Founder Structure to Published and Ongoing Studies

As of October 2009, the OOA studies at UM-SOM included approximately 4,800 participants. 30% of these participants studied are descended entirely from a subset (numbers of founders per individual ranged between 27 and 82) of the 201 founders born before 1800. That is, the entire gene pool of those individuals was derived from founders born before 1800. 99% of participants studied are descended entirely from a subset (numbers of founders ranged between 21 and 110) of the 255 founders born before 1900. A subset of 127 founders with more than 50 living descendants comprises the entire gene pool of 90% of the study participants. A subset of 102 founders with more than 100 descendants comprises the entire gene pool of 77% of the study participants.

### Excess of Female Founders

The number of female founders (332) is larger than the number of male founders (222) among 554 founders. We evaluated three possible causes for the excess of female founders: 1) remarriages of male founders after widowhood; 2) marriages comprising one founder and one non-founder; 3) possible overestimate of female founders due to the difficulty of tracking relationships between females with different married surnames.

We mentioned previously that there are 136 marriages between 136 female founders and 131 male founders. In addition to the 136 married founder couples, there are 196 female founders married to non-founder males, and there are 91 male founders married to non-founder females. Thus, remarriage of widowed male founders is only a minor factor in the excess of female founders. The propensity for female founders to marry non-founders is a more substantial factor, but this may be confounded by difficulty in correctly determining the founder status of females.

The lack of knowledge about relationships among female founders is partly due to the male bias in the FFH and AAMG books. Among 332 female founders, only 263 have partial names given, and only 207 have surnames, making it difficult to track relationships. There are 168 distinct surnames using identical spelling, and 159 after grouping some sets such as {Moser, Mosser, Musser} that are likely to be different spellings of the same surname. If any individuals with the same surname share ancestry, we would be able to reduce the number of female founders.

Occasionally, the books hint at a hidden relationship among founders, via footnotes, but the footnotes were not used systematically as they are often speculative. We conclude that the excess of female founders is mostly due to cryptic or unknown relationships, so that some of the apparent female founders are not really founders.

## Discussion/Conclusions

With new queries and utility programs in PedHunter version 2.0, we identified 34,160 presumed living OOA individuals born in 1930-2000 and connected them into a 14-generation pedigree descended from 554 founders, after trimming. Because of the small number of founders, the frequency of consanguineous marriages in OOA steadily increased over time and reached approximately 85% for individuals born in 1940-1959. Among consanguineous marriages, the median kinship coefficient stayed stable in the 19th century, but rose in the 20th century. Table 4 reports kinship coefficients of married OOA couples who had at least one offspring in AGDB4. Numbers of couples and kinship coefficients are both increased in temporal statistics.

**Table 4:** Distribution of birth years and kinship coefficients among married OOA couples with offspring in AGDB4.

Birth year	#(couples)	Average	25% percentile	Median	75% percentile
**1866-1875**	98	0.0244	0.0164	0.0258	0.0315
**1876-1885**	113	0.0272	0.0164	0.0258	0.0350
**1886-1895**	161	0.0272	0.0183	0.0267	0.0363
**1896-1905**	184	0.0286	0.0194	0.0292	0.0382
**1906-1915**	245	0.0289	0.0222	0.0309	0.0374
**1916-1925**	366	0.0300	0.0218	0.0297	0.0387
**1926-1935**	467	0.0317	0.0241	0.0318	0.0402
**1936-1945**	739	0.0310	0.0241	0.0326	0.0392
**1946-1955**	1,103	0.0315	0.0247	0.0319	0.0396
**1956-1965**	1,390	0.0335	0.0274	0.0344	0.0408
**1966-1975**	1,731	0.0352	0.0296	0.0352	0.0414
**1976-1985**	607	0.0363	0.0302	0.0359	0.0416

**Union set**	7,273	0.0326	0.0259	0.0333	0.0402

Birth year cohorts of ten years are reported in rows. A couple is assigned to the interval containing the average of the two birth years. Numbers of married OOA couples #(couples) and kinship coefficients are reported in columns. Couples born later tend to have larger kinship coefficients.

Analysis of cumulative mean RFR shows that 128 founders accounted for over 95% of the average founder contribution among all living OOA descendants. Such results confirm that the OOA in Lancaster County are truly a closed population. The combination of lack of new genetic material, lack of socio-economic variation, and detailed genealogies make the OOA ideally suited for identifying some rare variants that are associated with complex phenotypes [20]. The examples of GWAS replications cited in Background prove that some trait-associated SNP alleles seen in other populations can also be found in the OOA. Our characterization of the founder structure provides an explanation for why other trait-associated alleles did not enter the OOA population; for example, cystic fibrosis is one of the most common recessive diseases in individuals of European ancestry, but hardly seen among the OOA [57].

That the OOA have a small number of founders, but linkage disequilibrium (LD) patterns similar to outbred European populations [51] may seem paradoxical. The apparent paradox is explained by noting that LD is a measure of non-random association of alleles at different loci. Linkage between the loci maintains this association under random mating and drift, while recombination will decay the association. Thus, allele and haplotype frequencies drift together. For example, suppose two SNPs have D' = 1 in the founders, so that only three of the four possible haplotypes are observed, with frequencies 0.15, 0.35 and 0.5, respectively, but drift to 0.20, 0.40 and 0.40 in the current population. Both allele and haplotype frequencies have changed, but LD has not changed. The assumption of common alleles is important as random drift over the 14 generations spanned by AGDB will not eliminate common variation. For low frequency alleles there is longer LD in the Amish due to linkage as shown in [51] and also in some of our association studies [20,58], where the most significant signal can be more than 500 kbp from the causal allele.

One weakness in our characterization of the OOA founder structure is the apparent excess of female founders. We tested three hypotheses and concluded that the excess is mostly due to cryptic or unknown relationships, such that some female founders are not really founders. Analyses of mitochondrial genomes could provide evidence regarding some of these cryptic relationships. The male lineage and Y chromosome inheritance have been validated [59] a unique Y chromosome STR haplotype bred true within each of the 28 male lineages represented in the UM-SOM study sample. The 27 surnames of the males comprising these lineages captured 98% of the households in the 1998 Lancaster County Amish Address Book, which contains 42 distinct surnames. This number is much lower than the 222 total male founders because of genetic drift, conversion of some earlier settlers to other Anabaptist sects, and westward migration [35].

One limitation of our RFR calculations is that the variance of the kinship coefficient due to Mendelian sampling variance is not included in the calculation. For example, the kinship coefficient is 0.25 for both parent-offspring and full sibs, but the variance is zero for parent-offspring and non-zero for full sibs. Analytical expressions of kinship variances are not possible for complex pedigrees, but empirical variances can be obtained through simulation by repeated gene-dropping of distinct founder alleles through the pedigree. Computationally efficient gene-dropping can be achieved using the ancestor-first pedigree renumbering described above. We may implement a function *r-empirical *in a future version of PedHunter.

The notion of "founder" we used is with respect to a trimmed genealogy, not including any explicit conditions on the locations of birth or death. The data sources focus on individuals who lived at least some years in the present United States, but not exclusively. To quantify this, we looked at the source information on 220 founders with known or estimated birth years in and before 1755, and we found that 81 founders (44 females and 37 males) probably died in Europe or on the sea. Therefore, there may be even more female founders who never reached North America.

To increase the utility of AGDB and other digitized genealogies, we continue to enhance PedHunter with queries and utility programs to assist the discovery process on pedigrees in large genealogies. For example, AGDB has been used to estimate the encatchment population of specific hospitals to determine the denominator of the hip-fracture incidence [60]. For a study of osteogenesis imperfecta, AGDB was used prospectively to identify individuals likely to carry a genetic variant of phenotypic interest [61]. A pedigree constructed from AGDB made it possible to trace the origin of a rare *APOC3 *null allele conferring a favourable lipid profile and apparent cardioprotective phenotype to a couple born at the turn of the 19^th ^century [20]. Additionally, PedHunter has been used in other pedigrees, to discover genetic drift and founder effects [62].

In sum, we quantified the founder structure of the OOA and implemented numerous improvements to the software PedHunter that will be useful in future studies of both the OOA and other populations with large, computerized genealogies.

## Abbreviations

AAMG: Amish and Amish Mennonite Genealogies; AGDB: Anabaptist Genealogy Database; FFH: Fisher Family History; GWAS: genome-wide association studies; IBD: identical-by-descent; LD: linkage disequilibrium; OOA: Old Order Amish; RFR: relative founder representation; UM-SOM: University of Maryland School of Medicine.

## Competing interests

The authors declare that they have no competing interests.

## Authors' contributions

W-JL implemented the queries added in PedHunter version 2.0, carried out the analysis of founder contributions and AGDB shown here, and wrote the paper. TIP developed the method for analyzing founder contributions, analyzed the founder contributions using an earlier version of AGDB with the PEDSYS package [53], suggested additions to PedHunter, and wrote parts of the paper about founder contributions. JRO helped develop the method for founder contributions and suggested additions to PedHunter. RA implemented most of the queries added in PedHunter versions 1.1 through 1.3 and organized data for all versions of AGDB including version 4.0 and FISHER formally announced in this paper. AAS conceived AGDB and PedHunter, assisted in their implementation, and supervised the project. All authors edited the paper, read and approved all submitted versions including the final manuscript.

## Pre-publication history

The pre-publication history for this paper can be accessed here:

http://www.biomedcentral.com/1471-2350/11/68/prepub

## Supplementary Material

Additional file 1 PedHunter 2.0 queries and utility programs. Click here for file
