# Supplementary material for: PedHunter 2.0 and its usage to characterize the founder structure of the Old Order Amish of Lancaster County
Source: BMC Med Genet. 2010 May 2;11:68. doi: 10.1186/1471-2350-11-68 (PMC2880975; doi:10.1186/1471-2350-11-68)
Supplement: Additional file 1 — PedHunter 2.0 queries and utility programs. [file 1471-2350-11-68-S1.PDF]

**Testing a relationship**	**Finding individuals**	**Printing information**	**Complex query**	**Utility**
is_father	*spouses*	*person_info*	*family*	*linkage2tables*
is_mother	*spouses_file*	*children_info*	*subset*	*verify_tables*
is_child	*father*	*children_couple_info*	*all_shortest_paths*	*generations*
is_sibling	*mother*	*family_info*	*all_shortest_paths_count*	*subped*
is_half_sibling	*children*		*kinship*	*print_pedigree*
is_first_cousin	*siblings*		*inbreeding*	*renumber_pedigree*
is_ancestor	*half_siblings*		*ancestors_ped*	*trim_pedigree*
is_descendant	*uncles_aunts*		*descendants_ped*	
is_founder	*first_cousins*		*all_relatives*	
	ancestors		*acp*	
	ancestors_file		*asp*	
	descendants		*average_r*	
	descendants_file		*calculate_r*	
	lca		*minimal*	
	lca_file			
	birth_death			
	age			
	living			
	lifespan			
	founder			
	founder_birth			
	founder_descendant			
	count_descendant			
